# Review of Self-Powered Wireless Sensors by Triboelectric Breakdown Discharge

**DOI:** 10.3390/mi16070765

**Published:** 2025-06-29

**Authors:** Shuzhe Liu, Jixin Yi, Guyu Jiang, Jiaxun Hou, Yin Yang, Guangli Li, Xuhui Sun, Zhen Wen

**Affiliations:** Jiangsu Key Laboratory for Carbon-Based Functional Materials & Devices, Institute of Functional Nano and Soft Materials (FUNSOM), Soochow University, Suzhou 215123, China; 2114401098@stu.suda.edu.cn (S.L.); 20224214160@stu.suda.edu.cn (J.Y.); 2214401098@stu.suda.edu.cn (G.J.); 2214401081@stu.suda.edu.cn (J.H.); 2214401046@stu.suda.edu.cn (Y.Y.); 2214401090@stu.suda.edu.cn (G.L.); xhsun@suda.edu.cn (X.S.)

**Keywords:** triboelectric nanogenerator (TENG), breakdown discharge, wireless sensing, RLC circuit model, signal analysis

## Abstract

This review systematically examines recent advances in self-powered wireless sensing technologies based on triboelectric nanogenerators (TENGs), focusing on innovative methods that leverage breakdown discharge effects to achieve high-precision and long-distance signal transmission. These methods offer novel technical pathways and theoretical frameworks for next-generation wireless sensing systems. To address the core limitations of conventional wireless sensors, such as a restricted transmission range, high power consumption, and suboptimal integration, this analysis elucidates the mechanism of the generation of high-frequency electromagnetic waves through localized electric field ionization induced by breakdown discharge. Key research directions are synthesized to enhance TENG-based sensing capabilities, including novel device architectures, the optimization of RLC circuit models, the integration of machine learning algorithms, and power management strategies. While current breakdown discharge sensors face challenges such as energy dissipation, multimodal coupling complexity, and signal interpretation barriers, future breakthroughs in material engineering and structural design are anticipated to drive advancements in efficiency, miniaturization, and intelligent functionality in this field.

## 1. Introduction

**Figure 1:**
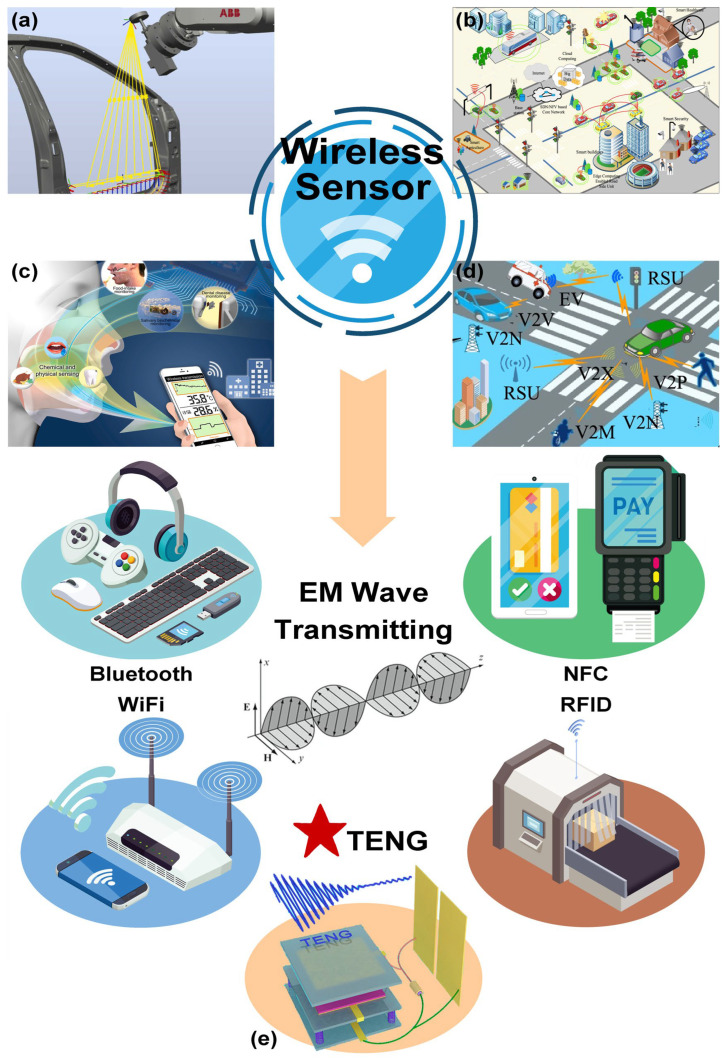
Typical application scenarios of wireless sensors and main implementation paths of wireless signal transmission. Application of wireless sensors in (**a**) intelligent manufacturing [4]; (**b**) smart cities [6]; (**c**) medical health [7]; (**d**) intelligent transportation [8]. (**e**) TENGs provide a new development idea for wireless sensor technology paths [26].

Wireless sensors represent a critical component of Industry 4.0, with their data measurement and analysis capabilities playing a pivotal role in advancing industrial automation and lifestyle intelligence, making them indispensable for enhancing production and operational efficiency [1,2]. Wireless sensors have demonstrated extensive applications across domains such as smart manufacturing [3,4], smart cities [5,6], healthcare [7], and intelligent transportation [8] (Figure 1a–d). Compared with traditional wired sensors, wireless sensors stand out because of their ease of installation, deployment flexibility, and cost-effectiveness [9]. The inherent scalability of triboelectric breakdown discharge-based sensing and its compatibility with real-time data transmission protocols render this technology particularly suitable for dynamic and complex environments. As the Internet of Things (IoT) proliferates and integration requirements escalate, wireless sensors drive the interconnection, digitization, and intellectualization of industrial processes through functionalities such as automated data collection, multidimensional monitoring, data aggregation, and remote control, thereby showing substantial development potential [10].

Traditional wireless sensing systems rely predominantly on wireless local area networks (WLANs), such as Wi-Fi and Bluetooth technologies, to establish wireless connections among devices within specific areas [11]. While Wi-Fi achieves longer transmission distances at the cost of a higher power consumption, Bluetooth conversely provides a lower power consumption but with limited communication range [12,13]. As active systems, WLANs involve numerous components and often face challenges such as an excessive physical size and insufficient integration levels when applied to distributed sensing platforms [14]. On the other hand, passive signal transmission systems such as NFC and RFID still struggle with short transmission distances [15,16,17,18]. Therefore, developing a wireless sensing platform capable of balancing transmission distance, power consumption, and system integration has remained a persistent research focus.

Electromagnetic waves serve as fundamental carriers for wireless signal transmission. When an electric current varies, it generates a changing electric field in its vicinity. This varying electric field, in turn, induces a time-varying magnetic field in an orthogonal direction. The cyclic interplay between these electromagnetic fields propagates electromagnetic waves [19,20]. In conventional radio transmission systems, high-frequency alternating currents are typically generated by oscillatory circuits. These currents are excited through coils to generate alternating electromagnetic fields, thereby forming radio waves [21,22]. Similarly, in triboelectric nanogenerators (TENGs), charge transfer processes produce fluctuating currents and voltages, creating the necessary conditions for the emission of electromagnetic waves [23,24,25,26]. As shown in Figure 1e, the TENG-driven self-powered wireless sensing paradigm, with the capability of converting the ambient energy of mechanical movements into electrical signals, has provided a new pathway for wireless sensors.

Recently, the triboelectric breakdown discharge principle has been proposed to utilize the high voltage triggered by TENGs for signal transmission. This new mechanism critically relies on a localized electric field amplification at sharp electrode tips, where accumulated triboelectric charges exceed the gas breakdown threshold, thereby generating transient plasma channels that emit intense electromagnetic pulses. Such a breakthrough fundamentally overcomes the inherent limitations of conventional displacement current-based wireless transmission in both signal intensity and propagation distance. This review systematically examines key developments in wireless sensing driven by the triboelectric breakdown discharge mechanism since 2021, providing a multidimensional analysis spanning discharge mechanisms, device architectures, algorithmic integration, and system-level optimization. Our synthesis aims to establish a robust theoretical framework and actionable technical pathways for advancing self-powered wireless sensing technologies.

## 2. Working Mechanism of TENGs in Wireless Sensing

**Figure 2:**
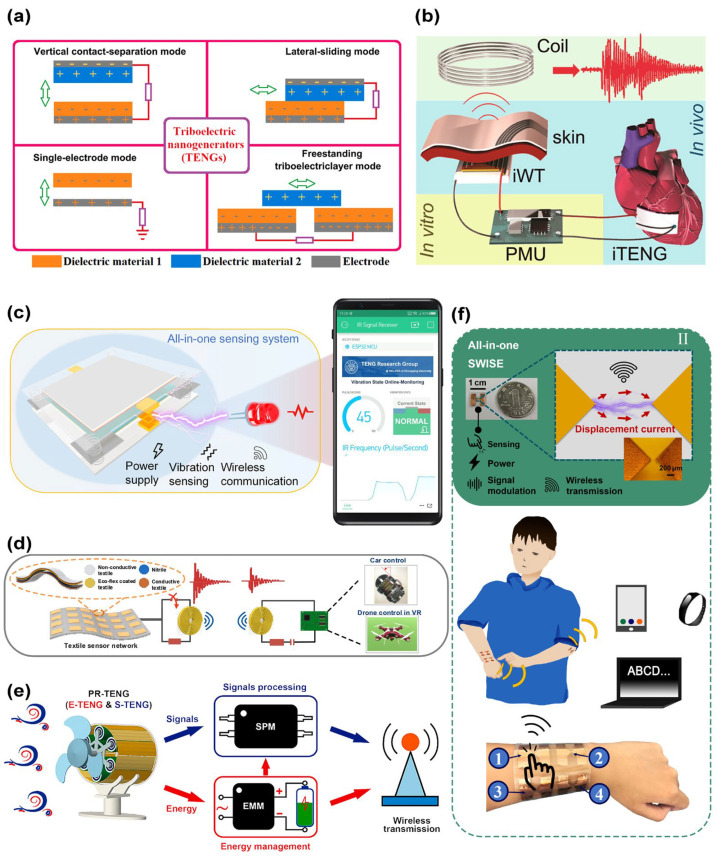
Working principle of TENGs and various technical routes used by TENG for wireless sensing. (**a**) Four working modes of the TENG [28]. (**b**) Implantable TENG for wireless heart monitoring in vivo [43]. (**c**) TENG-driven infrared wireless communication sensing system for vibration monitoring [29]. (**d**) Short-distance self-powered wireless sensor network based on a TENG direct sensing mechanism [41]. (**e**) Wind-driven wireless anemometer based on rolling contact [38]. (**f**) Full self-powered long-distance wireless sensing scheme based on a discharge-induced displacement current [49].

The triboelectric nanogenerator (TENG) was first proposed in 2012 [27]. It utilizes two materials with distinct triboelectric series, which generate triboelectric charges through relative sliding under mechanical deformation or displacement. This process, driven by the coupled effects of contact electrification and electrostatic induction, enables the conversion of mechanical energy into electrical energy. TENGs can harvest diverse forms of mechanical energy through four fundamental operational modes: vertical contact–separation mode, lateral sliding mode, single-electrode mode, and freestanding triboelectric layer mode (Figure 2a) [28].

TENGs are capable of harvesting ambient energy and converting subtle, irregular mechanical motions into electrical signals. These signals can then be transmitted via visible or infrared light to convey state parameters (Figure 2c) [29,30,31,32,33]. Alternatively, TENGs demonstrate versatility as self-sustained power sources for external radiofrequency (RF) devices such as Bluetooth and Wi-Fi modules, enabling wireless signal transmission while addressing the energy supply challenges in wireless sensor networks (Figure 2e) [34,35,36,37,38]. In addition, TENGs can function directly as signal acquisition devices by detecting mechanical input parameters such as vibrations, wind speed, displacement, and flow rate (Figure 2b) [39,40,41,42,43]. The integration of TENGs with resonant circuits allows the generation of electromagnetic wave signals through emitters such as coils or planar electrodes, leveraging the transient discharge characteristics of microswitches or diodes. This enables short-range wireless power transfer or frequency-modulated signal transmission for applications, including electronic device charging, data sensing, and remote control [44,45,46,47,48]. However, the existing sensing methods have the following challenges. Sensing systems utilizing intrinsic displacement currents for EM wave emissions are fundamentally constrained by short transmission ranges. Meanwhile, optical signal transmission methods suffer from modulation complexity, and transmission relying on external RF modules inevitably increases systems’ complexity while reducing their energy conversion efficiency.

Recently, a novel sensing solution has been proposed that utilizes the breakdown discharge triggered by the accumulation of triboelectric charge to induce the generation of electromagnetic waves (Figure 2f) [49,50]. The mechanism of electromagnetic waves’ emission via breakdown discharge primarily involves gas mediums’ ionization under high-voltage conditions. At critical voltage thresholds, free electrons in the gas acquire sufficient energy from the electric field. These energized electrons then collide with gas molecules, initiating molecular ionization. This cascade process leads to electron-ion multiplication and the formation of a plasma channel discharge [51]. During this process, rapid charge movement and the establishment of the plasma channel generate intense electromagnetic field fluctuations, thereby producing electromagnetic waves [52]. The breakdown voltage threshold follows Paschen’s law, which describes a nonlinear relationship between the gas breakdown voltage and the product of the gas pressure (p) and electrode separation distance (d) [53,54,55]. Through sharp-tip discharge configurations, the air breakdown voltage can be reduced to the range of 100–1000 V [56]. Given that TENGs inherently exhibit high-voltage, low-current characteristics, their perfect alignment with these voltage requirements establishes them as ideal candidates for efficient electromagnetic wave excitation.

In TENG systems, the triboelectric effect induces the accumulation of a charge on materials’ surfaces through frictional contact between two objects, governed by the principles of electrostatic induction. By strategically guiding the localized accumulation of static charges, such as adding a discharge tip or a gas discharge tube in the circuit, the resultant electric field intensity becomes significantly amplified. When this enhanced local field strength surpasses the breakdown threshold of the gaseous medium, a controlled discharge occurs. Through the inductance and capacitance components preset in the circuit, the current signal can be oscillated and amplified, generating high displacement currents that facilitate wireless electromagnetic signals’ emission and transmission [57]. Through the breakdown discharge principle, TENGs can transmit wireless signals without requiring external signal transmission units. Furthermore, key signal parameters such as frequency and amplitude attenuation can be precisely regulated through circuit components, effectively bypassing traditional communication encoding/decoding processes.

In wireless self-powered sensing systems utilizing the triboelectric breakdown discharge mechanism, external mechanical inputs are converted into electromagnetic wave signals through the coupled effects of triboelectrification and controlled breakdown discharge. This process entirely bypasses the external power modules essential for conventional sensing systems, thereby achieving self-powered capability. The system’s versatility is demonstrated through adaptable mechanical input methods such as wind energy, fluid-driven actuation, or human movement, ensuring broad applicability across diverse scenarios. A comparison of wireless sensors using different energy harvesting methods and signal exciting mechanisms is shown in Table 1. This breakthrough technology, which leverages a triboelectric charge accumulation-induced breakdown discharge for the generation of electromagnetic waves, has exceptional advantages, including compact form factors, a high integration density, extended transmission ranges, and superior scalability.

## 3. Advances in Triboelectric Breakdown Discharge Applications in Wireless Sensing

### 3.1. Device Design and Sensing Mechanisms

**Figure 3:**
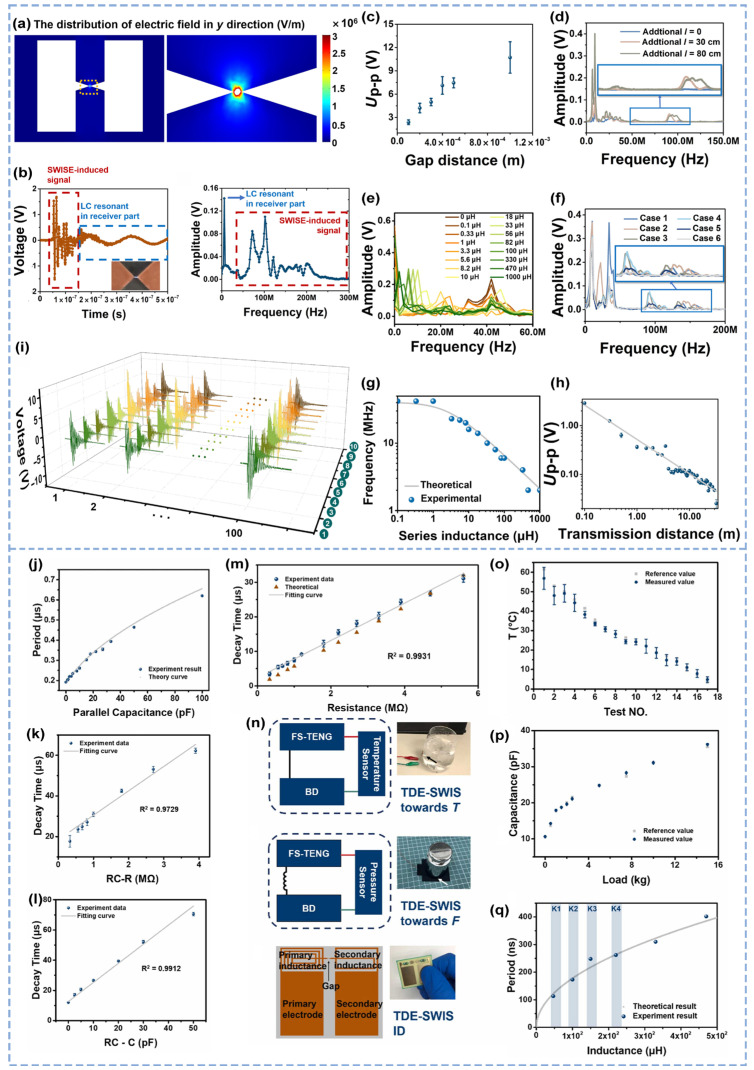
Signal characteristics, parameter coupling, and application verification of the TENG breakdown discharge wireless sensing platform [49,50]. (**a**) COMSOL simulation analysis of the electric field distribution of the SWISW device in the breakdown state. (**b**) Time domain and frequency domain signal characterization of the SWISE excitation signal. (**c**–**h**) Effects of external factors on the breakdown discharge signal, including (**c**) discharge tip gap, (**d**) wire length, (**e**,**g**) inductance, (**f**) spatial conductor distribution, and (**h**) receiver distance. (**i**) Signal output of the multigas breakdown dose response. (**j**) The change in the signal period under the regulation of parallel inductance. (**k**) Influence of resistance on decay time under resistance-inductance regulation and (**l**) influence of capacitance on decay time. (**m**) Variation in attenuation time under the control of series resistance. (**n**) Commercial sensors and the coupling mode between ID identification and TDE-SWIS. (**o**) Sensing temperature. (**p**) Comparison of the sensing weight with the reference value. (**q**) Comparison between the signal periods of components with different inductors and the theoretical values for ID identification.

Recent advancements have led to the development of a self-powered wireless sensing e-sticker (SWISE) based on the generation of a displacement current through breakdown discharge (Figure 2f) [49]. When activated, a SWISE generates negative charges in its triboelectrically charged layer. Under electrostatic induction, an electric field forms between electrodes, with the highest field intensity concentrated around sharp tips, as shown by the COMSOL simulation result in Figure 3a, triggering a controlled breakdown discharge. This process produces rapidly varying displacement currents, which in turn generate high-frequency signals. Wireless signals captured by oscilloscopes undergo Fourier-transform analysis, revealing spectral distributions primarily in the very high frequency (VHF) band, whereas receiver resonance frequencies center at approximately 10 MHz (Figure 3b). The signal reception characteristics are influenced by multiple factors, including the wire length, spatial conductor distribution, receiver distance, discharge tip geometry, and inductance (Figure 3c–h). SWISEs demonstrate multifunctional capabilities such as gas species discrimination, motion detection, and wireless keyboard interfacing. By collecting 100 repeated datasets from each of 10 distinct gas environments for training and testing, with each waveform containing 2500 data points, a SWISE achieved a gas classification accuracy of 98.5% through extracting unique breakdown discharge signatures across gas environments using a bi-directional long short-term memory (Bi-LSTM) model (Figure 3i). Adjustments to SWISEs’ structural parameters enable amplitude/frequency modulation, allowing discrimination between different keystroke signals. Integrated with oscilloscopes and MATLAB (R2020a)-based signal processing, this enables applications such as self-powered wireless keyboards and electronic skins for human motion tracking (Figure 2f). Notable for its ultracompact form factor (thickness: ≤95 μm, size: 9 × 9 mm^2^, mass: 16 mg) and mechanical flexibility, the SWISE achieves a wireless transmission range of 30 m. These attributes establish it as a groundbreaking solution for next-generation self-powered sensing platforms, particularly in wearable and IoT applications where miniaturization and energy autonomy are critical. The SWISE exhibits an ultrathin profile (thickness: ≤95 μm), compact dimensions (9 × 9 mm^2^), lightweight construction (mass: 16 mg), and mechanical flexibility, while achieving a wireless transmission distance of 30 m. These attributes collectively establish it as an innovative paradigm for self-powered wireless sensing platforms, particularly in space-constrained and dynamic deployment scenarios.

While the SWISE pioneered single-device multifunctionality through discharge signal modulation, its fixed-circuit architecture limited its cross-parameter sensing capabilities. To address this constraint, the research team further integrated triboelectric discharge effects with commercial sensors, developing a more versatile triboelectric discharge effect-enabled self-powered wireless sensing solution (TDE-SWIS) system capable of the high-precision monitoring of multiple physical parameters, including temperature and pressure [50]. The system’s equivalent circuit can be modeled as a resistor–inductor–capacitor (*RLC*) tuned circuit, with tunable parameters encompassing the natural frequency *ω_n_* and damping ratio *ζ*, which are the inherent parameters of the circuit system.

(1) $$\omega_{n}=\sqrt{\frac{1}{LC}}$$

(2) $$\zeta =\frac{R}{2\sqrt{\frac{L}{C}}}$$

These critical parameters are dynamically adjustable through a strategic modulation of the circuit’s resistive, inductive, and capacitive components. The natural frequency *ω_n_* mainly affects the fundamental frequency (*f*_0_), and the damping ratio *ζ* principally changes the relative attenuation time (*T*). By adjusting resistor–inductor–capacitor (*RLC*) variables, *ω_n_* and *ζ* are affected, thus changing *f*_0_ and *T*, so the wireless signal can be modulated by *RLC* parameters. Under series external resistor (*R_s_*) regulation or resistor-capacitor (*RC*) hybrid regulation, parameter modulation primarily affects the relative attenuation time (*T*) of intermediate-frequency signals induced by circuit oscillations, where *T* is defined as the time required for the system response to decay to 1/e of its initial value. For series resistor regulation, the relationship between *T* and the external resistance *R_s_* is as follows:

(3) $$\tau =R_{s}C_{0}+\alpha$$

where α is the adjustment constant. The experimental results demonstrate that when *R_s_* ranges from 220 kΩ to 100 MΩ, *T* increases monotonically with resistance, with fitted curves exhibiting a high linearity and close alignment with theoretical predictions (Figure 3m). For *RC* hybrid regulation, the *T-RC* relationship is governed by

(4) $$\tau =R(C_{0}+C_{p}+C_{pa})+\alpha$$

With a fixed parallel capacitance *C_p_* = 10 pF, *T* increases linearly and monotonically with resistance (Figure 3k). Conversely, when the parallel resistance *R_p_* = 1 MΩ, *T* increases linearly with capacitance (Figure 3l). In inductor–capacitor (*LC*) regulation, parameter adjustments predominantly modulate the signal’s fundamental frequency (*f*_0_), which is calculated via

(5) $$f=\frac{1}{2\pi \sqrt{\left(L_{s}+L_{0})(\frac{C_{0}C_{s}}{C_{0}+C_{s}}\right)}}$$

under series conditions and

(6) $$f=\frac{1}{2\pi \sqrt{\left(L_{s}+L_{0})(C_{0}+C_{p}\right)}}$$

under parallel conditions. Here, *f*_0_ increases monotonically with increasing capacitance, and the experimental results strongly agree with the theoretical curves (Figure 3j). For validation in application, the system integrates diverse commercial sensors (Figure 3n). When a commercial thermistor-based temperature sensor is coupled with the TDE-SWIS system, hand motion-induced mechanical inputs generate temperature-dependent *T* variations: *T* increases as the cup-wall temperature decreases, matching reference values (Figure 3o). In *LC*-regulated pressure sensing, a capacitive pressure sensor (100 μH inductor) achieves pressure measurements with only 0.18% error relative to reference values (Figure 3p). Additionally, multinode sensing is realized by integrating distinct inductors with miniaturized TDE-SWIS units, enabling wireless signal differentiation across devices through frequency modulation (Figure 3q). These advancements significantly enhance cross-physical signal compatibility, confirming the versatility of the triboelectric discharge platform for multiparameter sensing applications.

### 3.2. Algorithm Fusion and Multimodal Sensing

**Figure 4:**
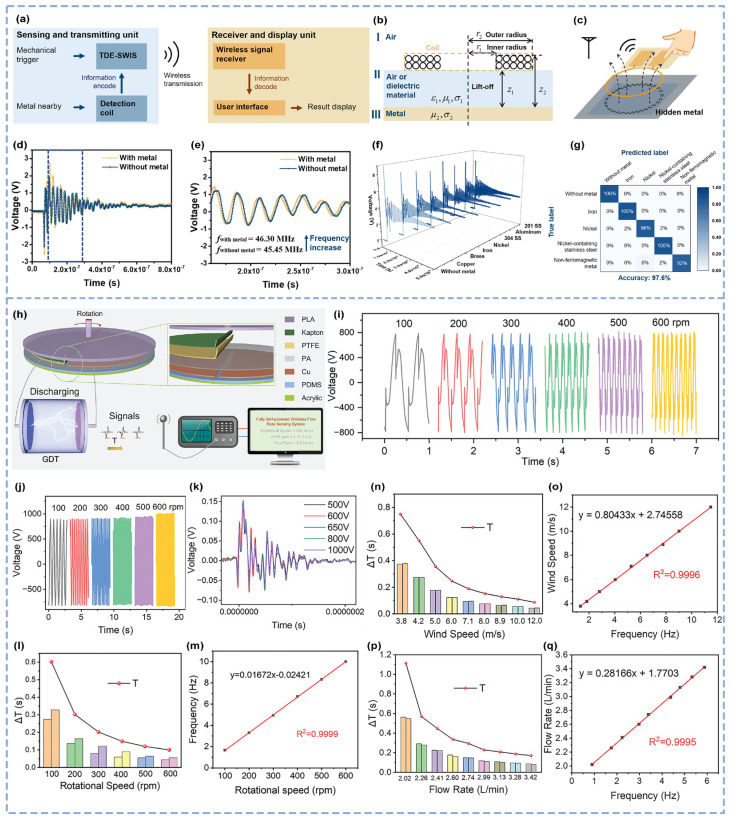
A self-powered wireless metal detector combining a CNN and a fluid mechanics-oriented self-powered wireless sensor flowmeter [59,60]. (**a**) SWMD device workflow and receiving system. (**b**) Physical model of the eddy current coil for metal detection. (**c**) Mechanistic demonstration based on the eddy current reverse magnetic field cancellation effect. (**d**) Contrast analysis of the received signal when the metal is static and (**e**) local enlargement of the picture. (**f**) Polymetallic TDE-SWIS time domain signal and (**g**) CNN metal classification confusion matrix. (**h**) Schematic diagram of the R-Teng structure and signal transmission process. (**i**) Voltage characteristics of GDT breakdown at different rotating speeds. (**j**) *V_oc_* of the R-TENG at different speeds. (**k**) Time domain diagram of signals generated by different *V_oc_* values during breakdown discharge at 400 V GDT. Linear relationship between (**l**) ΔT and (**m**) frequency with the speed of continuous discharge at different speeds. Linear relationship between (**n**) ΔT and (**o**) frequency with the flow rate of continuous discharge for gas fluid sensing. Linear relationship between (**p**) ΔT and (**q**) frequency with the flow rate of continuous discharge for liquid-fluid sensing.

The circuit parameter modulation strategies validated in TDE-SWIS provided critical insights for subsequent research. Capitalizing on these advancements, researchers integrated inductive coupling effects with convolutional neural networks (CNNs) to address metal detection requirements, developing a self-powered wireless metal detector (SWMD) based on the modulation of coil inductance by external metals (Figure 4a,b) [59]. Figure 4b shows the electrical model of the metal detection coil. When mechanical input is applied to the device, breakdown discharge is triggered to generate electromagnetic wave signals. When a metal object approaches the coil, the induced time-varying magnetic field is perturbed, generating currents within the metal. According to Lenz’s law, the magnetic field in the metal opposes the changing magnetic field induced in the coil, thereby altering the coil’s impedance (Figure 4c). The equivalent electrical model of the metal detector is represented as a series combination of a variable inductor and resistor. Parameters such as the distance, position, and type of adjacent metal influence the coil’s inductance through inductive coupling, modulating the electromagnetic waves (Figure 4d,e). The frequency test results demonstrate minimal frequency deviations in the measurements caused by nonmetallic, nonferromagnetic, and ferromagnetic interferences at the detection frequency (Figure 4f). Using a CNN for auxiliary identification, signals without metal and those containing seven metal types were measured. The dataset was divided into five groups (without metal, non-ferromagnetic metals, pure iron, pure nickel, and nickel-containing stainless steel), with a total sample size of 1000. A confusion matrix was constructed using an image-based algorithm, and the recognition results from training and testing demonstrated high positive predictive values and true positive rates, achieving an overall classification accuracy of 97.6% (Figure 4g). In practical tests, the compact detector successfully identified brass plates concealed within books and effectively monitored metal objects activated by footstep-induced triggers. These validations confirm the high sensitivity and reliability of the self-powered wireless metal detector in real-world applications.

Having established the efficacy of breakdown discharge in solid-state sensing, researchers further explored its potential in dynamic fluid systems. Building on the stable excitation of triboelectric breakdown discharge beyond threshold voltages, the platform was extended to fluid monitoring, yielding a compact-sized, fully self-powered wireless sensing flowmeter (CSWF) for real-time gas and liquid flow velocity measurements (Figure 4h) [60]. Driven by a rotating TENG (R-TENG), charges accumulate between the electrodes of a gas discharge tube (GDT), forming an alternating high-voltage field. When the voltage reaches the GDT threshold, instantaneous breakdown discharge occurs across the air gap, generating rapidly varying displacement currents and high-frequency signals (Figure 4i). By calculating the breakdown discharge period, the rotational speed can be derived. The experimental results reveal a robust linear relationship (R^2^ = 0.9999) between rotational speed and discharge frequency *(f* = 1/*T*), achieving stable signal transmission over distances beyond 10 m (Figure 4l,m). The output voltage remains largely unaffected by the rotational speed and saturates at 800 V (Figure 4j). The GDT consistently triggers breakdown discharge each time the output voltage reaches the threshold (*Vth*), confirming the excitation stability of the flow sensor. Time domain and frequency domain analyses reveal a significant consistency in electromagnetic wave signal trends, with frequency distributions primarily below 300 MHz, validating the sensor’s immunity to environmental interference (Figure 4k). For gas flow measurement (CSWG), a tunable wind turbine simulates natural wind conditions, with anemometer cups mechanically linked to the R-TENG’s rotating shaft for energy harvesting. The gas flow velocity and discharge frequency exhibit a strong correlation coefficient of 0.9996 (Figure 4n,o). For liquid flow measurement (CSWG), a water wheel connected to the shaft harvests energy from ambient water flow, achieving a correlation coefficient of 0.9995 between the liquid flow velocity and discharge frequency (Figure 4p,q). These results confirm the exceptional accuracy of the CSWF in monitoring gas and liquid flow velocities, as well as the applicability of the breakdown discharge-based RLC circuit model in hydrodynamic scenarios.

**Figure 5:**
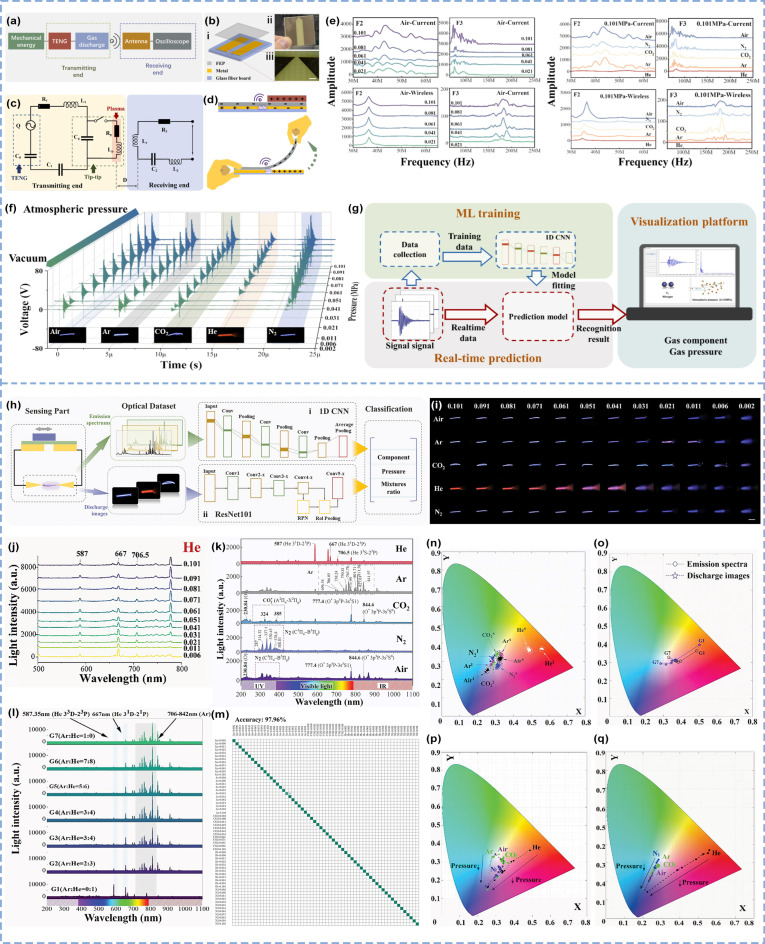
TENG breakdown discharge gas sensing via electromagnetic signal analysis and optical emission analysis [61,62]. (**a**) Self-driving wireless electronic nose system framework. (**b**) Structure diagram of the integrated transmitting unit: i. The structure diagram. ii. The physical image. iii. The enlarged image of the discharge tips. (**c**) Equivalent circuit model of the wireless sensor system. (**d**) Schematic diagram of the sliding mode and peeling mode of the integrated transmitter. (**e**) Current signal and wireless signal in the sensing characteristic frequency domain in the air pressure identification and atmosphere identification modes. (**f**) Some 3D images of typical wireless signals of triboelectric discharge in 60 different atmospheres. (**g**) Working process of machine learning gas identification. (**h**) Workflow of light emission gas sensing. (**i**) Friction-induced discharge images at different atmospheres and pressures. (**j**) Emission spectra of He gas discharge at different pressures. (**k**) Emission spectra of gas discharges in different atmospheres. (**l**) Emission spectrum of mixed gas discharge. (**m**) Identification confusion matrix for 60 kinds of gas environment based on the emission spectrum. (**n**) CIE XY chromaticity diagram of discharge for different gas types. (The five-pointed star with “S” is the spectral data, and “I” is the image data.) (**o**) CIE XY chromaticity diagram of discharge under different gas mixture ratios. (Empty dots and black dotted lines are spectral data, and hollow five-pointed stars and blue dotted lines are image data.) (**p**) CIE XY chromaticity diagram of discharge images under different air pressures. (**q**) CIE XY chromaticity diagram of the discharge emission spectrum under different air pressures.

By leveraging mature CNN algorithms, the triboelectric discharge platform has been further extended to gas sensing applications. The research team achieved the dual identification of gas species and pressure by not only utilizing electromagnetic signal spectral features (Figure 5a) but also analyzing triboelectric discharge-generated optical emission spectra and luminescent images (Figure 5h). The TENG and gas discharge components were integrated into a centimeter-scale chip (Figure 5b), and its electrical model—encompassing mechanical input, gas discharge, signal transmission, and reception—is illustrated in Figure 5c. Gas discharge can be generated by triggering the device through sliding or peeling motions (Figure 5d). Owing to differences in the critical ionization energy and mean free path among gas molecules, discharge signal analysis enables the effective identification of gas atmospheres (Figure 5f) [61]. The gas atmosphere exerts dual effects on the oscillatory discharge current at the transmitter: the breakdown voltage *V_b_* influences the amplitude of the discharge current, whereas the capacitance, inductance, and resistance of the gas affect the resonant frequency and attenuation time. Spectral signal analysis reveals that the low-frequency region (0–30 MHz) corresponds to the system’s intrinsic frequency, where peak frequencies remain unaffected by gas species, but their amplitudes vary with gas type and decrease as the gas pressure decreases. In the 30–65 MHz and 65–250 MHz bands, variations in gas species affect both the amplitude and frequency of peaks, whereas pressure variations influence only peak magnitudes (Figure 5e). For identification testing, a 1D convolutional neural network (Figure 5g) was trained on 30,000 samples covering 5 gas types and 12 pressure levels (totaling 60 distinct atmospheres), enabling the system to simultaneously recognize gas species and pressure with a mean accuracy of 93.8%.

In the optical emission-based identification of gases, triboelectric discharge generates high-output voltages at the kilovolt (kV) level, producing optical emissions that include emission spectra and discharge images (Figure 5i–l), which are influenced by gas species and pressure (Figure 5n–q) [62]. By utilizing a sliding freestanding triboelectric-layer mode TENG (SFT-TENG) to produce stable high voltages (±10 kV) and charges (±400 nC), gas discharge-induced optical emissions are generated in sealed chambers via breakdown discharge. Machine learning algorithms analyze the features of emission spectra and discharge images to identify single gas species, gas mixtures, and gas pressures. Emission spectral signals are processed through a 1D convolutional neural network (1D CNN). For single-gas and pressure identification, the training dataset comprised 12,000 samples covering 5 gas types (Air, Ar, CO_2_, He, and N_2_) and 12 pressure levels, achieving a detailed identification accuracy of 97.96% (Figure 5m). For the recognition of gas mixtures, the system attained an accuracy of 96. 46% across seven mixed gases. Discharge images are analyzed via a ResNet-101 architecture, using a dataset of the same size, yielding identification accuracies of 98.18% for the 5 gas types and 97.34% for the 12 pressure levels, with a gas mixture sensing model accuracy of 98.43%.

### 3.3. Energy Management and System Optimization

**Figure 6:**
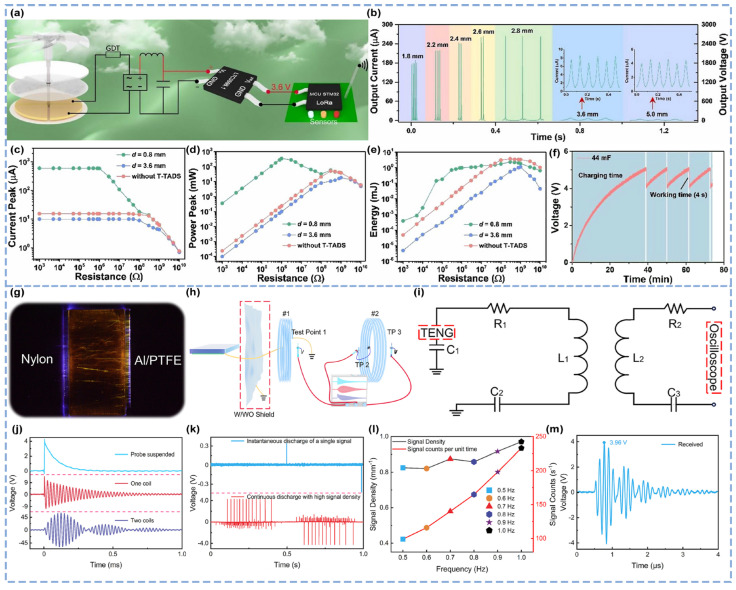
The Teng-driven tip electrode air discharge switch was used in meteorological sensing, and high-signal-density wireless sensing coupled with a coil was used [58,63]. (**a**) Schematic diagram of a self-driven wireless weather sensing system equipped with a tip electrode air discharge switch. (**b**) Measurement results of the output current and voltage increasing with the discharge gap (1.8–5.0 mm). (**c**) Instantaneous output current; (**d**) peak power value; (**e**) output energy per cycle. (**f**) Curve of the 44-MF capacitor charging wireless weather sensing system in PMC mode. (**g**) Discharge photos of the HSD sensor during operation. (**h**) Schematic diagram of the shielding detection of primary coil #1 in the air breakdown state. (**i**) Equivalent circuit model of a wireless transmission scheme integrating a TENG and a pair of induction coils. (**j**) Signal details of three detection modes: probe suspension, probe connected to a single coil, and probe connected to a secondary coil pair at a detection distance of 20 cm. (**k**) Comparison of the signal density between traditional wireless TENG sensors and HSD sensors. (**l**) Signal density (black broken line) and signal count per unit time (red broken line) of the equipment at a driving frequency of 0.5–1.0 Hz. (**m**) The image received by the oscilloscope at the receiving end at a driving frequency of 1 Hz, a pressure of 40 N, and a distance of 100.69 m.

To address energy dissipation in TENGs’ operation, researchers have integrated a triboelectric breakdown discharge switch with a power management circuit (PMC), developing a TENG-driven tip–tip electrode air discharge switch (T-TADS) that significantly enhances TENGs’ output performance and PMCs’ energy storage efficiency (Figure 6a) [63]. Compared with mechanical and electronic switches, gas discharge switches exhibit a superior vibration resistance, wear tolerance, interference resistance, rapid response, lightweight design, and ease of integration. The integration of TENGs with gas discharge switches typically results in two discharge modes: arc discharge and corona discharge. When the T-TADS is closed, the TENG generates output currents and voltages of 15.5 μA and 155.4 V, respectively. In arc discharge mode (discharge gap ≤ 2.8 mm), multiple pulsed discharge peaks occur per cycle. At a discharge gap of 0.1 mm, the instantaneous output current and voltage peaks reach 58.2 μA and 582.4 V, respectively, with these peaks increasing as the discharge gap widens (Figure 6b). In corona discharge mode (discharge gap > 2.8 mm), the instantaneous output current and voltage peaks decrease sharply. The impedance matching analysis reveals that the maximum output power is achieved only when the external resistance equals the T-TADS equivalent impedance (~1 MΩ for arc discharge, ~100 MΩ for corona discharge). With an external resistance of 1 kΩ in arc discharge mode, the instantaneous output current peak (Figure 6c) and power peak (Figure 6d) increase by approximately 40 times and 1600 times, respectively, compared with those of the TENG without a T-TADS. Energy-per-cycle measurements confirm that the peak output energy of the arc discharge mode surpasses that of the conventional TENG when the resistance is less than 30 MΩ (Figure 6e). However, the maximum output energy in arc discharge mode remains lower than that of the TENG without T-TADS because of energy dissipation as light and sound during discharge. Using the air discharge switch, a self-powered wireless meteorological sensing system was constructed, comprising a rotating freestanding triboelectric-layer TENG, PMC, voltage regulator, and signal transmitter. A wind-driven TENG charges the capacitor in the PMC. The initial charging takes 39 min, and then the charging time between working stages is 11 min. This system transmits temperature, humidity, pressure, and illuminance data every 11 min, achieving a maximum transmission distance of 800 m (Figure 6f).

Beyond energy management optimization, to investigate the feasibility of breakdown discharge sensing in complex dynamic processes, researchers have coupled triboelectric breakdown discharge with inductive coils, achieving high-density, high-energy, long-distance wireless signal sensing (Figure 6h) [58]. Breakdown discharge primarily occurs between the PTFE/nylon and Al electrodes (Figure 6g), generating hundreds of recordable discharges within a single sliding cycle (Figure 6k). When two inductive coils are connected to the discharge circuit and signal testing probe, the electrical model corresponds to an RLC circuit (Figure 6i), which includes two inductors. The resonant gain induced by the RLC circuit significantly amplifies the signal amplitude. The signals formed by the superposition of oscillatory components with different frequencies and phases exhibit decaying oscillations with multiple envelopes, thereby extending the sensing range (Figure 6j). The signal density is markedly increased (Figure 6l), enabling the acquisition of approximately 311 high-amplitude signals (3.96 V) under a driving frequency of 1 Hz, a 40 N pressure, and a wireless transmission distance of 100.69 m (Figure 6m). This breakthrough in signal density advancement allows for the analysis of smaller sliding intervals, providing a foundation for the fine-grained resolution of microscale mechanical interactions.

**Figure 7:**
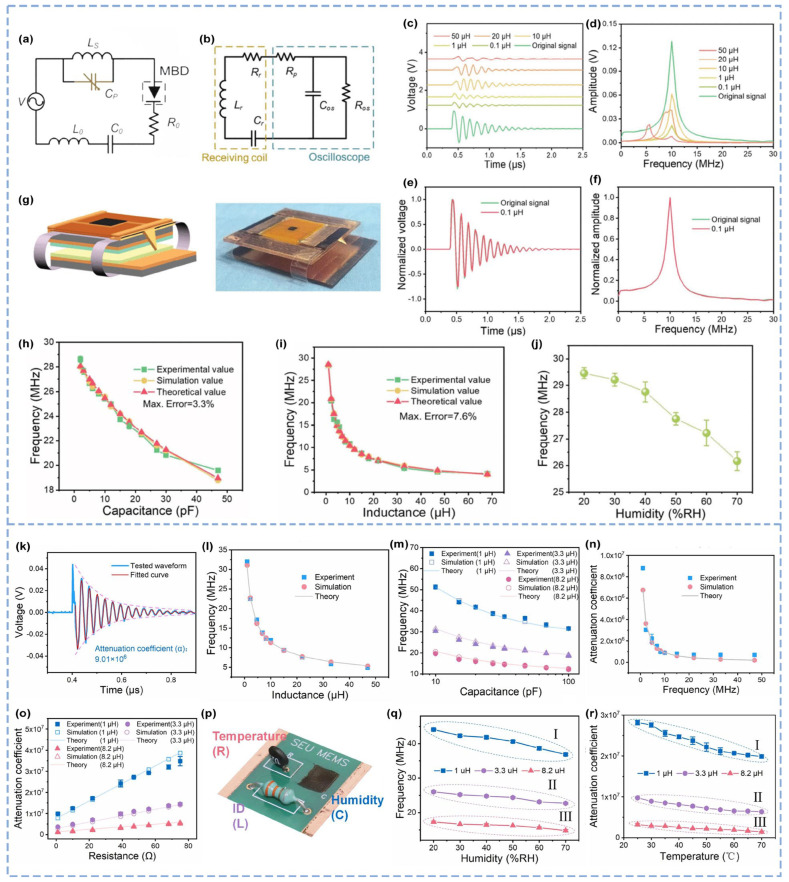
A laminated TENG with an integrated sensing module and breakdown discharge tip and a dual-mechanism sensing scheme based on the frequency and attenuation coefficient [64,65]. (**a**) Circuit model of the wireless sensor. (**b**) Circuit model of the receiver. (**c**) Time and (**d**) frequency domain diagrams of signals measured by an oscilloscope with different inductances. (**e**) Time and (**f**) frequency domain diagrams of the original signal and the received signal with a 0.1 μH inductance. (**g**) Structural schematic and photograph of the wireless humidity sensor. (**h**) Relationship between the base frequency and series inductances. (**i**) Relationship between the base frequency and parallel capacitance. (**j**) Relationship between the base frequency and humidity. (**k**) Fitting results of the attenuation coefficient for a typical wireless signal. (**l**) Relationships between the base frequencies and series inductances. (**m**) Relationships between the base frequencies and series capacitances at 1, 3.3, and 8.2 μH. (**n**) Relationships between the attenuation coefficients and series inductances. (**o**) Relationships between the base frequencies and series resistances at 1, 3.3, and 8.2 μH. (**p**) Photograph of the PCB-based multiparameter sensing module. (**q**) Base frequency versus humidity curves of wireless signals with different series inductances. (**r**) Attenuation coefficient versus temperature curves of wireless signals with different inductances.

Discharge sensing systems have been extensively validated in the aforementioned studies; however, challenges persist in the complexity of wireless signals’ modulation and demodulation. Another study addressed this issue by theoretically analyzing circuit responses and wireless signal waveforms during breakdown discharge, simulating the impact of different receiver coils on signal reception, optimizing signal reception fidelity, and fabricating a laminated TENG integrating sensing and discharge modules [64]. This system was successfully demonstrated in humidity sensing applications. The wireless sensor’s circuit model is shown in Figure 7a. During breakdown discharge, the circuit response can be modeled as an equivalent resistor-inductor-capacitor (*RLC*) series circuit. According to Kirchhoff’s voltage law, the current in the circuit satisfies

(7) $$L\frac{di}{dt}+Ri+\frac{1}{C}\int idt=u_{i}(t)$$

where *u_i_*(*t*) denotes the voltage signal generated by the TENG. During breakdown discharge, the voltage instantaneously drops to zero, and the circuit response exhibits underdamped oscillatory decay with a characteristic frequency:

(8) $$i(t)=\frac{1}{L\omega_{n}\sqrt{1-\zeta^{2}}}e^{-\zeta \omega_{n}t}\sin(\omega_{n}\sqrt{1-\zeta^{2}t})$$

where *ω_n_* is the natural frequency, and *ζ* is the damping coefficient. The receiver, comprising a detection coil and oscilloscope, can also be modeled as an *RLC* circuit (Figure 7b). The coil size and winding count affect its inductance, parasitic capacitance, and resistance, thereby influencing damping oscillations. The oscilloscope captures a composite signal combining the native wireless signal and the coil’s damped oscillations. On the basis of electric field intensity calculations, the received wireless signal is expressed as

(9) $$\varepsilon_{0}(t)=Be^{-\zeta \omega_{n}t}\sin(\omega_{n}\sqrt{1-\zeta^{2}}t+\gamma )$$

where *B* and *γ* are constants. The oscillation frequency is given by

(10) $$f=\frac{\omega_{n}\sqrt{1-\zeta^{2}}}{2\pi }=\frac{1}{2\pi \sqrt{LC}}\sqrt{1-\zeta^{2}}=\frac{1}{4\pi }\sqrt{\frac{4L-R^{2}C}{L^{2}C}}$$

Since the damping factor ζ≪1 in practical devices, the frequency simplifies to

(11) $$f=\frac{\omega_{n}\sqrt{1-\zeta^{2}}}{2\pi }=\frac{1}{2\pi \sqrt{LC}}\sqrt{1-\zeta^{2}}\approx \frac{1}{2\pi \sqrt{LC}}$$

The study utilized Cadence 2019 simulation software to analyze the impact of receiver coils with different inductances on wireless signal reception, employing exponentially decaying sinusoidal signal sources to emulate real-world wireless transmissions. The simulation results demonstrated that larger inductors amplify damping oscillations in the receiver system, complicating composite signals and hindering wireless signal extraction (Figure 7c,d). In contrast, smaller inductors effectively attenuate RLC oscillations and better restore the original electromagnetic waveform (Figure 7e,f). The laminated TENG design integrating sensing and breakdown discharge modules (Figure 7g) comprises a movable upper electrode with a sharp-tip structure—featuring a 60° triangular copper foil attached to the side of the upper copper layer—and a fixed lower electrode. The two modules are interconnected via a PET elastic structure, enabling complete tip to lower copper contact and discharge initiation under compressive force. Signal modulation was validated through theoretical calculations, simulations, and experiments. By integrating commercial series inductance *L_s_* and parallel capacitance *C_p_*, the experimental results align with the simulation outputs and theoretical formulas, confirming the feasibility of the wireless sensing model and signal modulation (Figure 7h,i). For validation in application, graphene oxide (GO) was incorporated as a humidity-sensitive material whose capacitance varies with ambient humidity. As the relative humidity (RH) increased from 20% to 70%, the base frequency exhibited a pronounced shift toward lower frequencies (Figure 7j), demonstrating the system’s ability to characterize humidity-dependent signals.

Building on this foundation, researchers have proposed a distance-independent self-powered multimechanism wireless sensing scheme (SMWSS) based on the attenuation coefficient and base frequency mechanisms [65]. This study analyzed the receiver’s current response waveform and explored a novel wireless sensing modulation mechanism utilizing attenuation coefficients combined with frequency modulation to decouple the sensing parameters through two independent mechanisms—the attenuation coefficient and base frequency—resulting in a wearable multiparameter sensor. In SMWSS, the circuit response is modeled as a zero-state response under step signal excitation, where the receiver’s current response is expressed as

(12) $$i_{R}(t)=Ce^{-\alpha t}\sin(\omega_{n}t+\psi )=Ce^{-\alpha t}\sin(2\pi ft+\psi )$$

where *f* represents the base frequency, which is derived from the periodic signal variation and identified as the dominant spectral peak via fast Fourier transform (FFT), and *α* denotes the attenuation coefficient:

(13) $$\alpha =\zeta \omega_{n}=\frac{R}{2L}$$

which determines the signal amplitude decay rate and is calculated by fitting the oscillatory decay region in the time domain waveform (Figure 7k). Adjusting the *RLC* parameters in the sensing module to couple wireless signals revealed that increasing the series inductance prolongs the oscillation period, shifting the base frequency to lower values (Figure 7l); increasing the series capacitance reduces the base frequency across different inductances (Figure 7m); and increasing the series resistance does not interfere with the oscillation period but accelerates signal attenuation. The attenuation coefficient (*α*) varies significantly with the inductance (Figure 7n) and shows a linear relationship with the resistance at a fixed inductance (Figure 7o) while remaining unaffected by the capacitance. The experimental, simulated, and theoretical results demonstrated a strong overlap in the *f*-modulating and *α*-modulating curves. Although the received signal amplitude decreases with transmission distance, *f* and *α* remain invariant, confirming distance-independence. Leveraging the independence between the *f-α* modulating mechanisms, a multiparameter module was designed (Figure 7p), integrating inductors for ID recognition, thermistors for temperature sensing, and humidity-sensitive capacitors. Capacitance and resistance changes exclusively modulate *f* and *α*, enabling humidity and temperature measurements without cross-interference, whereas inductors define ID-specific base frequency ranges. For humidity sensing, three inductance IDs were calibrated, as shown in Figure 7q. For temperature sensing, corresponding attenuation coefficient ranges were established, as shown in Figure 7r. The system allows personalized sensing targets via the modular replacement of resistive/capacitive components and scalability through additional inductors. Proof-of-concept applications—including obstacle-penetrating wireless manipulators and battery-free hopping robots—demonstrate SMWSS’s potential in smart homes and robotics. By implementing a dual-modal *f-α* decoupling strategy, this work establishes a precise multiphysical sensing framework for self-powered systems, enabling the synchronous monitoring of diverse parameters.

## 4. Discussion

Self-powered wireless sensing technology based on triboelectric breakdown discharge has made remarkable progress in recent years (Table 2). Its core breakthrough lies in the use of the high-voltage output characteristics of a triboelectric nanogenerator (TENG) to excite electromagnetic wave signals through breakdown discharge, thus overcoming the inherent contradiction between the transmission distance, power consumption, and integration of traditional wireless sensing systems. The RLC circuit parameter control strategy skips the complex mechanism of the traditional WLAN coding layer and establishes a quantitative mapping relationship between physical quantities and signal characteristics, which provides a theoretical basis for multiphysical quantity sensing. At the application level, the breakdown discharge mechanism demonstrates a remarkable adaptability to different scenarios. The pressure monitoring achieved by integrating commercial sensors with the discharge system, as well as precise gas–liquid flow velocity measurements, validates its practicality in industrial monitoring. Furthermore, the fusion of this technical pathway with deep learning algorithms has expanded its application boundaries to gas species identification and metal classification.

These advances have proven the universality of the breakdown discharge platform in cross-scale and multiphysical quantity sensing, but its technical maturity is still limited by several key challenges, including reducing energy loss, simplifying signal analysis, diminishing environmental interference, and enhancing the device’s durability. Addressing these challenges, the unique advantages of wireless sensing technology through triboelectric breakdown discharge in terms of passivity, miniaturization, and the synchronous monitoring of multiple physical quantities could unfold a new blueprint through its application in intelligent manufacturing, environmental awareness, and wearable devices.

### 4.1. Reducing Energy Loss

The problem of energy loss in the process of breakdown discharge has not been completely solved. As shown in Figure 6e, the T-TADS system exhibits lower maximum output energy than the TENG without a switch. This energy loss arises from light and heat dissipation during arc discharge. Previous researches have established a theoretical model from the perspective of the dynamic balance of friction charge and expounded various mechanisms and improvement methods of friction charge attenuation [66]. The energy output performance can be improved by the optimization of materials, power management, charge pumps, and other technologies [34]. Researchers have put forward a cascade capacitor breakdown model for the dynamic output of DC-TENGs and defined, then adjusted, the possible discharge domain to realize the optimization of their output [67]. Different gas environments also have an impact on the output charge density of DC-TENGs [68]. By studying the dielectric properties and discharge plasma dynamics of this kind of material, the energy dissipation in the process of breakdown discharge can be potentially reduced, so as to obtain a larger output signal and signal-to-noise ratio.

### 4.2. Simplifying Signal Analysis

The complexity of signal analysis also constitutes a bottleneck in its application. For example, environmental sensing needs to address multiple characteristics, such as the base frequency and attenuation time, at the same time, which places higher requirements on real-time processing algorithms. The recognition process of determining base frequencies is indirect and may lead to incorrect results. The output current signal of TENGs has been studied and analyzed, and a weak current signal measurement method based on the I/V measurement conversion method is proposed, which converts a high-impedance weak-current signal into a low-impedance low-voltage signal [69]. Another study analyzed the retention time of the charge saturation state in TENG sensors and prepared a sensor whose output signal was not affected by departure time, by filling deep charge traps to improve the barrier and limit the surface charge generated [70].

The integration of machine learning algorithms has opened new pathways to overcome the limitations of conventional spectral analysis methods. As demonstrated in Figure 4f,g, time domain spectral analysis alone struggles to detect minute signal variations and exhibits large errors in the measurement of attenuation time, making it challenging to distinguish wireless signals carrying coupled metal information in SWMD systems. However, when a CNN was trained on the signal dataset, the system achieved an overall accuracy of 97.6% in metal identification. Triboelectric breakdown discharge sensing systems integrated with machine learning algorithms have demonstrated effectiveness in gas sensing and metal identification and are poised to expand into broader industrial applications. Through signal conversion or parameter limitation measurement methods, or in combination with machine learning algorithms, it is expected to see a reduction in the complexity of the signal analysis of triboelectric breakdown discharge sensors, providing a basis for the simpler coupling of sensing objects and the sensing with more parameters.

### 4.3. Diminishing Environmental Interference

Environmental sensitivity is another limiting factor. The signal interference between multiple coupled stimuli will affect the accuracy of the sensor, such as the fundamental frequency drift of humidity sensors under the influence of temperature. Researchers have used the independence between different response mechanisms (the thermionic emission effect and contact area effect) of pressure and temperature stimuli to propose a coupled but low cross-sensitivity bimodal response mechanism, which can clearly distinguish touch and temperature signals when the temperature and pressure change simultaneously [71]. The stability of materials and the packaging of the device also affect the consistency of sensor response in different environments. In addition to traditional metal and polymer materials, composite textile materials have been widely used in TENGs because of their sensitivity and flexibility [72]. Materials, interface design, and antijamming coupling technology need to be further optimized to meet the requirements of triboelectric sensors in different working environments.

### 4.4. Enhancing Device Durability

The material stability of triboelectric dielectrics and the integrity of their micro-nano architectures critically determine the operational robustness of TENGs under complex operational conditions, directly governing their energy conversion efficiency and service lifetime. As mechanically driven energy converters, TENGs inherently subject their triboelectric layers and electrodes to sustained mechanical stresses or deformations during prolonged operation, making material durability and structural integrity paramount. Factors including the aging of materials, wear from cyclic contact friction, and environmental variables such as temperature and humidity fluctuations collectively degrade the stability of triboelectric interfaces [41]. Especially in triboelectric breakdown discharge devices, it is often necessary to operate under the conditions of a high transient current and the repeated electric etching of tip materials. The fatigue wear of friction interfaces and the accumulating thermal effect on the discharge tip may lead to molecular chain fracture or lattice distortion, which may lead to energy output attenuation. By adding mesoporous silica-modified material into acrylate resin as a wear-resistant material, researchers have achieved a better antifriction performance and self-healing capability under heat treatment [73]. Furthermore, by using silicon-modified polyurethane (PU) as a self-repairing friction layer, the broken PU molecular chain can be gradually cross-linked by hydrogen bonds at room temperature to achieve a self-repairing effect [74]. The friction layer of zwitterionic polyurethane (Z-PU) can realize mechanical and electric breakdown self-repair based on an electrostatic interaction self-repair mechanism, which can be potentially applied to the discharge devices [75]. By studying the structural changes and chemical modification of the friction layer and discharge tip during wear, and using specific materials for optimization, the maintenance-free time of triboelectric breakdown discharge devices can be extended, paving the way for more mature applications.

## 5. Conclusions

In this paper, the research path of self-powered wireless sensing technology based on triboelectric breakdown discharge is systematically combined, and its core value in overcoming the bottlenecks of transmission distance, power consumption, and the integration of traditional wireless sensing systems is revealed. By summarizing innovative practices such as SWISE miniaturized patches, TDE-SWIS circuit coupling control, the metal/gas sensor CNN algorithm, and the fluid monitoring platform, the universality of the breakdown discharge mechanism in typical scenarios of wireless sensor networks is verified. At present, this technology still faces challenges, such as energy loss in the process of breakdown discharge, difficulty in coupling multimodal physical quantities, and the high complexity of multi-signal analysis. Significant potential remains for advancing triboelectric breakdown discharge systems, including the integration of novel materials and supersurface architectures to suppress the breakdown voltage. Multimodal data such as electromagnetic waves and optical spectra could be integrated to achieve accurate signal recognition for multiple sensing objects. Integrating and minimizing the RLC control circuit is expected to improve the compatibility of the sensing platform. In future research, further development of the triboelectric breakdown discharge mechanism in the era of interconnected intelligence will be realized through the optimization of materials and configurations, multi-channel signal identification, and circuit miniaturization.

## Figures and Tables

**Table 1:** Comparison of wireless sensors using different energy harvesting methods and signal exciting mechanism.

Reference	Energy Harvesting Method	Signal Exciting Mechanism	System Size (m^3^)	Transmission Distance (m)
[13]	External powered	Bluetooth	8.82 × 10^−7^	10
[17]	Wireless powered	NFC	5.25 × 10^−8^	2.5 × 10^−1^
[18]	Wireless powered	RFID	1.30 × 10^−7^	2.5 × 10^−2^
[29]	TENG self-powered	Optical signal	1.215 × 10^−4^	10
[45]	TENG self-powered	RFID	7.68 × 10^−7^	3 × 10^−3^
[46]	TENG self-powered	Coil	1 × 10^−5^	9 × 10^−1^
[26]	TENG self-powered	Planar electrode	1.25 × 10^−5^	2.3
[49]	TENG self-powered	Breakdown discharge	7.70 × 10^−9^	30
[58]	TENG self-powered	Breakdown discharge	1.6 × 10^−4^	100

**Table 2:** Comparison of different sensing applications of triboelectric breakdown discharge mechanism [49,50,58,59,60,61,62,63,64,65].

Sensing Solutions	TENG Mode	Sensing Objects	Recognition Methods	Transmission Distance
Self-Powered Wireless Sensing E-Sticker (SWISE)	Freestanding sliding TENG (FS-TENG)	ID recognition, gas sensing	Base frequency modulation, machine learning	~30 m
Triboelectric-Discharge Effect Enabled Self-Powered Wireless Sensing Solution (TDE-SWIS)	FS-TENG	Temperature, pressure, ID recognition	Base frequency modulation, decay time modulation	~30 m
Self-Powered Wireless Metal Detection (SWMD)	Contact–separation TENG (CS-TENG)	Metal recognition	Machine learning	/
Compact-Sized Fully Self-Powered Wireless Flowmeter (CSWF)	Rotating TENG	Fluid flow monitoring	Excitation frequency determination	~10 m
Self-Powered, Wireless, Multi-Dimensional Electronic Noses	Sliding freestanding triboelectric-layer TENG (SFT-TENG)	Gas sensing	Machine learning	40 cm (for test)
Self-Powered Multi-Dimensional Gas Sensing Solution	SFT-TENG	Gas sensing	Machine learning	/
TENG Driven Tip-Tip Electrode Air Discharge Switch (T-TADS)	Rotating freestanding triboelectric-layer TENG (FST-TENG)	Ambient meteorological data	External sensors	~800 m *
Integrated Wireless Sensing Scheme with High Signal Density (HSD)	Sliding TENG	High-density motion signals	Spectral analysis	~100 m
Self-Powered Multi-Mechanism Wireless Sensing Scheme (SMWSS)	Vertical CS-TENG	Coupling data of ID recognition, temperature, humidity	Base frequency modulation, attenuation coefficient modulation	~1.5 m

* T-TADS system uses the energy of TENG and discharge switch for Wi-Fi transmission.

## Data Availability

No new data were created or analyzed in this study. Data sharing is not applicable to this article.
